# An update on the regulatory mechanisms of NLRP3 inflammasome activation

**DOI:** 10.1038/s41423-021-00670-3

**Published:** 2021-04-13

**Authors:** Seungwha Paik, Jin Kyung Kim, Prashanta Silwal, Chihiro Sasakawa, Eun-Kyeong Jo

**Affiliations:** 1 Department of Microbiology, Chungnam National University School of Medicine, Daejeon, Korea; 2 Infection Control Convergence Research Center, Chungnam National University School of Medicine, Daejeon, Korea; 3 Medical Mycology Research Center, Chiba University, Chiba, Japan; 4 Nippon Institute for Biological Science, Tokyo, Japan

**Keywords:** NLRP3, inflammasome, inflammation, mechanism, interaction, Inflammasome, Autoimmunity, Infection

## Abstract

The NOD-, LRR-, and pyrin domain-containing protein 3 (NLRP3) inflammasome is a multiprotein complex involved in the release of mature interleukin-1β and triggering of pyroptosis, which is of paramount importance in a variety of physiological and pathological conditions. Over the past decade, considerable advances have been made in elucidating the molecular mechanisms underlying the priming/licensing (Signal 1) and assembly (Signal 2) involved in NLRP3 inflammasome activation. Recently, a number of studies have indicated that the priming/licensing step is regulated by complicated mechanisms at both the transcriptional and posttranslational levels. In this review, we discuss the current understanding of the mechanistic details of NLRP3 inflammasome activation with a particular emphasis on protein-protein interactions, posttranslational modifications, and spatiotemporal regulation of the NLRP3 inflammasome machinery. We also present a detailed summary of multiple positive and/or negative regulatory pathways providing upstream signals that culminate in NLRP3 inflammasome complex assembly. A better understanding of the molecular mechanisms underlying NLRP3 inflammasome activation will provide opportunities for the development of methods for the prevention and treatment of NLRP3 inflammasome-related diseases.

## Introduction

Inflammasomes are cytoplasmic high-molecular-weight protein platforms of caspase-1 activation in response to microbial invasion and damage signals.^1,2^ Inflammasomes consist of the nucleotide-binding oligomerization domain (NOD)-like receptor (NLR) family, the adapter apoptosis-associated speck-like protein containing a caspase recruitment domain (ASC), and the effector protease caspase-1. The formation of these protein complexes results in the activation of caspase-1, which is involved in the maturation of the proinflammatory cytokines interleukin-1β (IL-1β) and IL-18 into biologically active forms, and cleavage of gasdermin D (GSDMD) to promote pyroptotic cell death (pyroptosis).^3–6^

Among inflammasomes, the NOD-, leucine-rich repeat (LRR)-, and pyrin domain (PYD)-containing protein 3 (NLRP3) inflammasome has been studied extensively and was found to be activated by a wide spectrum of stimuli. It is generally accepted that NLRP3 inflammasome activation is regulated through a two-step process, with priming at the transcriptional and posttranslational levels (Signal 1) and assembly by multiple pathways in response to a variety of exogenous pathogen-derived or endogenous danger molecules (Signal 2). Recently, there has been a renaissance in our understanding of the posttranslational modification (PTM) and protein-protein interactions of NLRP3 inflammasome components that license cells for full activation of inflammasome assembly.^7–10^ The breadth of our current understanding extends to the regulation of the priming that is involved in NLRP3 inflammasome complex assembly, including accumulating evidence indicating a number of molecular mechanisms underlying the positive or negative regulation of NLRP3 inflammasome activation. Indeed, inflammasome and IL-1β activity are important for host defense against numerous bacterial, viral, and fungal infections. However, excessive or altered regulation of NLRP3 inflammasome activity is related to the pathogenesis of a wide variety of inflammatory, autoimmune, and degenerative diseases.^11,12^ The pleiotropic roles of the NLRP3 inflammasome have been reviewed extensively elsewhere in terms of physiological responses and in the context of a variety of human diseases.^13–16^ In addition, the mechanisms of noncanonical and one-step NLRP3 inflammasome activation are beyond the scope of this review.

Here, we summarize the current understanding of the molecular details involved in the priming/licensing step of NLRP3 inflammasome activation. We then cover the protein–protein interactions and spatiotemporal regulation of the NLRP3 inflammasome machinery. Finally, we discuss the various positive/negative regulatory mechanisms that orchestrate optimal regulation of the NLRP3 inflammasome.

### Overview of NLRP3 inflammasome activation

NLRP3 is an NLR that contains an N-terminal PYD, a central NAIP, CIITA, HET-E, and TP1 (NACHT) or NOD that hydrolyzes adenosine triphosphate (ATP) into adenosine diphosphate (ADP), and a C-terminal LRR domain. During inflammasome assembly, NLRP3 interacts with the N-terminus of the adapter protein ASC via PYD–PYD interactions; the C-terminus of ASC has a caspase recruitment domain (CARD) that can bind to procaspase-1 via CARD–CARD interactions to promote caspase dimerization and activation. Due to its prion-like properties, ASC forms large fibrillar aggregates known as “specks”.^11,12,17–20^

**Fig. 1:**
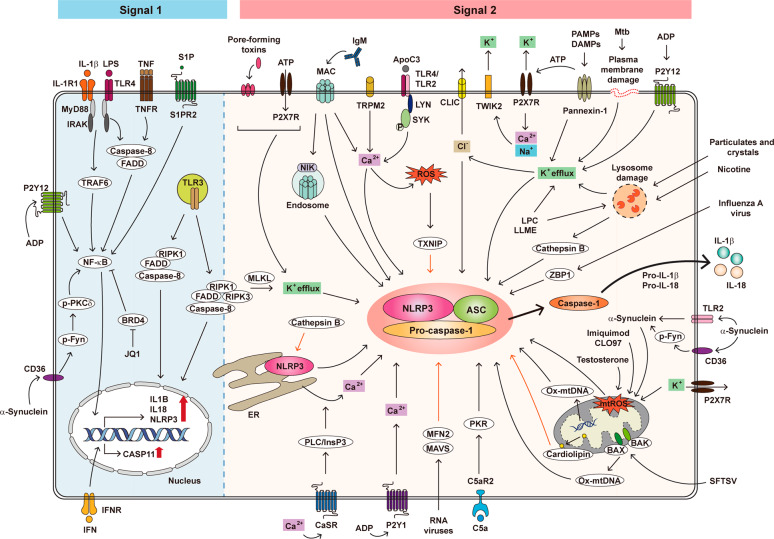
Overview of NLRP3 inflammasome priming and activation. NLRP3 inflammasome activation involves two steps, i.e., Signal 1 (priming) and Signal 2 (protein complex assembly). Signal 1 is triggered by pattern recognition receptor signaling or cytokines, leading to the transcriptional activation of NLRP3 inflammasome components. Licensing of the NLRP3 protein is important for the priming step of the NLRP3 inflammasome. The activation signal (Signal 2) is induced by various pathogen-associated molecular patterns (PAMPs) and damage-associated molecular patterns (DAMPs). Interleukin 1β (IL-1β)/IL-1R1, lipopolysaccharide (LPS)/Toll-like receptor 4 (TLR4), tumor necrosis factor (TNF)/TNF receptor (TNFR), sphingosine 1-phosphate (S1P)/S1P receptor 2 (S1PR2), adenosine diphosphate (ADP)/P2Y12, α-synuclein/CD36, and bromodomain-containing protein 4 (BRD4) inhibitor JQ1 each activate NF-κB and then upregulate the transcription level of the component required for NLRP3 inflammasome formation. Caspase-8 and fas-associated protein with death domain (FADD) are upstream regulators of NF-κB signaling that activate both the transcriptional priming and PTM of NLRP3 inflammasome pathway components. Upon TLR3 stimulation, FADD/caspase-8 scaffolding is involved in the PTM associated with Signal 1 in the intermediate pathway or activates receptor-interacting serine/threonine-protein kinases 3 (RIPK3)/mixed lineage kinase domain like pseudokinase (MLKL) function required for both Signal 1 and Signal 2 in the late pathway. Extracellular Ca^2+^ can activate the NLRP3 inflammasome through calcium-sensing receptor (CaSR), and CaSR triggers the phospholipase C (PLC)/inositol-1,4,5-trisphosphate (InsP3) pathway to induce intracellular Ca^2+^ release from the endoplasmic reticulum (ER). Ca^2+^ flux by transient receptor potential melastatin 2 (TRPM2) or apolipoprotein C3 (ApoC3) is mediated by reactive oxygen species (ROS) to activate NLRP3. It is currently recognized that TXNIP binds to NLRP3. ADP/P2Y1 induces Ca^2+^ movement, and various DAMPs/PAMPs trigger K^+^ efflux through pannexin-1 to activate NLRP3. In addition, P2X7 receptor (P2X7R) and tandem pore domains in weak inward rectifying K^+^ channel 2 (TWIK2) act as K^+^ efflux channels and are required for NLRP3 inflammasome activation. Testosterone, imiquimod, CLO97, K^+^ efflux, and α-synuclein generate mitochondrial ROS (mtROS), which activate the NLRP3 inflammasome. Severe fever with thrombocytopenia syndrome virus (SFTSV) infection triggers BCL2 antagonist/killer 1 (BAK)/BCL2-associated X (BAX) signaling and leads to oxidized mitochondrial DNA (ox-mtDNA). Furthermore, cardiolipin can directly bind to NLRP3 and activate NLRP3 inflammasome assembly. Particulates and crystals, nicotine, lysophosphatidylcholine (LPC), and Leu-Leu-O-methyl ester (LLME) induce lysosomal damage, and damaged lysosomes release cathepsin B. In addition, damaged lysosomes induce K^+^ efflux, which causes Cl^-^ efflux through chloride intracellular channels (CLICs). The complement system activates NLRP3 by forming a membrane attack complex (MAC). In endothelial cells, immunoglobulin M (IgM)-mediated MAC induces NF-κB-inducing kinase (NIK) stabilization and causes NLRP3 inflammasome activation. C5a-C5aR2 signaling also activates the NLRP3 inflammasome through protein kinase R (PKR) in macrophages. Moreover, pore-forming toxins and ATP induce K^+ ^efflux and activate NLRP3 inflammasome. During *Mycobacterium tuberculosis* (Mtb) infection, plasma membrane damage causes K^+^ efflux and NLRP3 activation. In addition, ADP also induces K^+^ efflux through P2Y12. During RNA virus infection, mitofusin 2 (MFN2) and mitochondrial antiviral signaling (MAVS) protein directly bind to and activate NLRP3. Cathepsin B also directly binds to NLRP3 in the ER. Z-DNA binding protein 1 (ZBP1) regulates NLRP3 activation in response to influenza A virus infection. Orange arrows indicate direct binding with NLRP3

NLRP3 inflammasome activation generally requires two steps, i.e., priming (Signal 1) and protein complex assembly (Signal 2) (Fig. 1). The priming process is triggered by pattern recognition receptor signaling, e.g., Toll-like receptor (TLR) 4 activation or tumor necrosis factor (TNF) signaling, which subsequently leads to the transcriptional activation of NLRP3, pro-IL-1β, and pro-IL-18 via nuclear factor-κB (NF-κB)-dependent pathways.^11,12^ However, emerging data suggest that the priming step of NLRP3 inflammasome activation is complicated, involving transcriptional and posttranslational mechanisms, and requires numerous protein binding partners.^11,12^ The activation signal (Signal 2) is induced by various pathogen-associated molecular patterns (PAMPs) and damage-associated molecular patterns (DAMPs), including extracellular ATP, pore-forming toxins, RNA viruses, and particulate matter. For Signal 2 activation, numerous molecular or cellular events, including mitochondrial dysfunction and reactive oxygen species (ROS) generation, ion flux (K^+^/Cl^−^ efflux, and Ca^2+^ influx), and lysosomal damage, are involved in the activation of NLRP3 inflammasome assembly.^11,12,17–20^ Both Signal 1 and 2 are triggered by microbial or sterile inflammatory stimuli, although microbial signals are different than sterile signals in terms of their kinetics and magnitude.^21^

In addition, NLRP3 inflammasome activation often leads to proinflammatory programmed cell death known as pyroptosis. The inflammasome complex leads to autoproteolytic activation of caspase-1, which subsequently triggers the cleavage of the proinflammatory cytokines IL-1β and IL-18 and the pyroptotic substrate GSDMD.^22,23^ Upon GSDMD cleavage, the N-terminus of GSDMD (N-GSDMD) oligomerizes and forms plasma membrane pores that mediate cell death and the secretion of the mature forms of IL-1β and IL-18.^22,23^ Recently, the Z-DNA binding protein 1 (ZBP1)-NLRP3 inflammasome, which is specifically activated by viral RNA products or endogenous nucleic acid ligands, was shown to promote a mixed form of cell death, i.e., pyroptosis, apoptosis, necroptosis (PANoptosis) and PANoptosome assembly.^24,25^ Another recent study revealed fundamental differences in GSDMD trafficking and cleavage between macrophages and neutrophils.^26^ In macrophages, N-GSDMD oligomerizes in the plasma membrane to form pores that promote NLRP3 inflammasome-associated pyroptosis.^27^ In contrast, N-GSDMD does not localize to the plasma membrane or mediate pyroptosis in NLRP3-activated neutrophils, although it is required for IL-1β secretion. Moreover, N-GSDMD associates with azurophilic granules to release neutrophil elastase into the cytosol and promotes secondary cleavage of GSDMD to form an alternatively cleaved form of N-GSDMD.^26^ We speculate that different NLRP3 inflammasome-activating stimuli in each cell type may determine the fate of cells through distinct patterns of GSDMD trafficking and cleavage. The regulatory mechanisms underlying pyroptosis and PANoptosis pathways are beyond the scope of this review. For information on the regulation of cell death during NLRP3 inflammasome activation, readers are directed to recent extensive and focused reviews in this area.^22–25,28^

### Signal 1: priming and licensing

It is generally thought that the priming process of NLRP3 inflammasome activation involves the transcriptional induction of NLRP3, as well as pro-IL-1β and pro-IL-18. However, a growing body of evidence suggests that the priming step involves more than transcriptional activation of NLRP3 to license its rapid activation toward Signal 2. Although the mechanisms of priming and licensing are not yet clear, the licensing of the NLRP3 protein is now generally accepted to be required for sufficient induction of functional NLRP3 by PTM regulation and the protein–protein interactions that enable the efficient assembly of inflammasome complexes. Investigations into these areas are rapidly expanding and have recently been extensively reviewed.^11,12^

While pro-IL-18 is constitutively expressed in monocytes and epithelial cells, it can also be induced by lipopolysaccharide (LPS) (TLR4), CpG oligonucleotides (TLR9), and the Sendai virus.^11,12,29^ TLR signals, such as TLR4/LPS, are the best known stimuli for transcriptional activation of pro-IL-1β, pro-IL-18, and NLRP3.^11,12,29^ Cytokines not involved in the inflammasome, such as TNF-α or type I interferon (IFN), enhance the priming process of inflammasome activation by influencing the transcription of inflammasome components.^11,29,30^ The mechanisms through which non-inflammasome proinflammatory cytokines contribute to priming/licensing remain to be characterized.

NF-κB signaling is essential for the transcriptional activation of priming responses to TLR and cytokine stimulation.^11,29–31^ Sphingosine 1-phosphate (S1P)/S1P receptor (S1PR) signaling is involved in the upregulation of NLRP3 priming through elevation of the gene expression of NLRP3 inflammasome components.^32^ Recently, the inhibition of bromodomain-containing protein 4 (BRD4), in the bromodomain and extraterminal domain (BET) family member of epigenetic readers, was reported to activate NF-κB signaling and enhance NLRP3 expression at the transcriptional level.^33^ Upon TLR/IL-1R signaling, TRAF6 is involved in the priming step of NLRP3 inflammasome activation through both transcriptional and nontranscriptional regulation of NLRP3.^34^ In addition, the TLR downstream adapter MyD88 and the IL-1 receptor-associated kinases IRAK-1 and IRAK-4 play crucial roles in the rapid activation of NLRP3 priming, presumably through PTM.^35–37^ These events lead to acute activation of caspase-1, regardless of new protein synthesis, thus suggesting that PTMs are crucial for the priming and licensing of the NLRP3 inflammasome.^11,29–31^

In addition, several reports show the critical role of fas-associated protein with death domain (FADD) and caspase-8 during the priming process of the NLRP3 inflammasome.^38,39^ FADD-caspase-8 plays an essential function in both canonical and noncanonical NLRP3 inflammasome activation through NF-κB–dependent transcription of pro-IL-1β and posttranslational activation of the NLRP3 inflammasome.^38^ Furthermore, FADD/caspase-8 scaffolding induces receptor-interacting serine/threonine-protein kinase (RIPK) 3/mixed lineage kinase domain-like pseudokinase (MLKL) activation required for both Signal 1 and 2 upon TLR3 stimulation.^39^ Together, these diverse intracellular signaling molecules, most are TLR-dependent, can prime the NLRP3 inflammasome at the transcriptional and posttranslational levels. Further work is needed to determine the precise mechanism by which different signaling molecules/pathways cooperate and cross talk during the transcriptional and posttranslational regulation of the priming/licensing process of canonical and noncanonical activation of the NLRP3 inflammasome.

In the next section, we discuss recent advances in our knowledge of NLRP3 interactions with molecular partners as well as several types of PTMs for NLRP3 priming/licensing, and Signal 2 is subsequently described.

### PTMs of NLRP3 and other components of the inflammasome complex

#### Ubiquitination

**Fig. 2:**
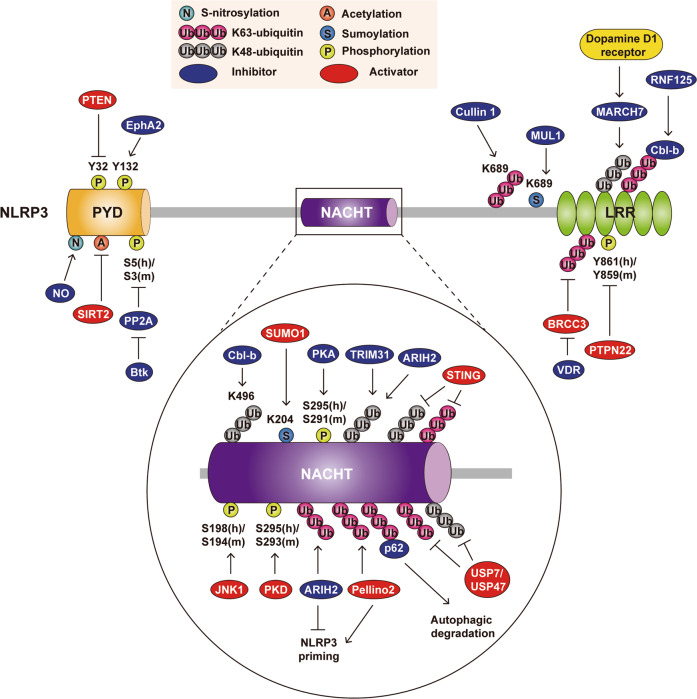
Posttranslational modifications (PTMs) in NLRP3 inflammasome components. The NLRP3 inflammasome is regulated by various PTMs, including ubiquitination/deubiquitination, phosphorylation/dephosphorylation, acetylation/deacetylation, SUMOylation, and nitrosylation, in different domains of NLRP3 (PYD, NACHT, and LRR). Activators and inhibitors of the NLRP3 inflammasome are represented by red and blue ovals, and phosphorylation sites of NLRP3 components from human and mouse species are indicated with (h) and (m), respectively.

Ubiquitination and deubiquitination of NLRP3 and other inflammasome components are essential for the assembly of the inflammasome complex (Fig. 2).^7^ Ubiquitination of NLRP3 by several E3 ligases is generally thought to abrogate inflammasome activation. Autophagic degradation of the NLRP3 inflammasome is mediated through K63 polyubiquitination of NLRP3 and subsequent interaction with the autophagic adapter p62.^40^ The E3 ubiquitin ligases RNF125 and Cbl-b are essential for targeting NLRP3 for K63- and K48-linked ubiquitination, respectively, ultimately leading to proteasome-mediated degradation.^41^

The E3 ligase TRIM31 binds and ubiquitinates NLRP3 for protein polyubiquitination and proteasomal degradation.^42^ In addition, dopamine-mediated inhibition of NLRP3 inflammasome activation is mediated through the E3 ligase MARCH7-mediated ubiquitination and degradation of the NLRP3 protein.^43^ Cullin1, the key component of the Skp1-Cullin1-F-box E3 ligase, interacts with NLRP3 and promotes the K63‐linked ubiquitination of NLRP3, in which K689 acts as a significant ubiquitin acceptor site in NLRP3.^44^ This ubiquitination of NLRP3 does not lead to its degradation but is crucial for the prevention of NLRP3 activation.^44^

Furthermore, Ariadne homolog 2 (ARIH2), the E3 ligase for binding and ubiquitinating NLRP3 at K48 and K63, is a negative regulator of NLRP3 priming activity in macrophages.^45^ However, another study showed that the E3 ubiquitin ligase Pellino2 is essential for NLRP3 ubiquitination during the priming step, thereby further promoting activation of the NLRP3 inflammasome.^46^ The precise mechanisms through which multiple E3 ligases and different sites in NLRP3 cooperatively or separately control NLRP3 inflammasome licensing remain to be fully determined.

#### Deubiquitination

Deubiquitination of NLRP3, another key process for the licensing of NLRP3 inflammasome activation, depends on TLR4 and mitochondrial ROS (mtROS) generation.^11,12,47^ Priming signals triggered through TLR4 or TLR2 stimulation leads to the induction of Abraxas brother protein 1 (ABRO1), a subunit of the BRCA2-containing complex subunit 3 (BRCC3, human BRCC36) deubiquitinase complex, thereby deubiquitinating the LRR domain of NLRP3 upon inflammasome activation.^47–49^ Notably, vitamin D receptor (VDR) appears to be a negative regulator of NLRP3 oligomerization and activation by blocking BRCC3-mediated deubiquitination of NLRP3.^50^ In the responses to nigericin and calcium pyrophosphate dihydrate (CPPD) crystals, both ubiquitin-specific peptidase (USP) 7 and USP47 function as essential deubiquitinating enzymes (DUBs) for the removal of ubiquitin from NLRP3 and inhibition of ASC speck formation. Both USP7 and USP47 have functional redundancy in deubiquitinating NLRP3, although ubiquitin linked to NLRP3 at K48 and K63 is not removed by USP7/USP47 upon inflammasome activation.^51^ Further studies to identify the precise targets for deubiquitination by USP7/USP47 in the context of licensing NLRP3 inflammasome activation are warranted.

Under conditions of cytosolic DNA stimulation and herpes simplex virus type 1 (HSV-1) infection, stimulator of interferon genes (STING) promotes NLRP3 inflammasome activation through recruitment and interaction with NLRP3 via attenuation of K48- and K63-linked polyubiquitination.^52^ Given that aberrant activation of the cGAS-cyclic GMP-AMP (cGAMP)-STING pathway leads to inflammation, senescence, and cancer,^53,54^ it is difficult to clarify the potential detrimental effects of the cGAS-STING pathway on NLRP3 licensing for inflammasome assembly. However, studies are beginning to identify the substrate-targeting mechanisms by which E1/E2/E3 ligases and DUBs regulate activation of the NLRP3 inflammasome. Given that current studies encompass only small numbers of E3s and DUBs in the regulation of NLRP3 priming, future structural and biochemical studies are warranted to reveal the functions and mechanisms of other currently uncharacterized ubiquitin ligases/DUBs in terms of NLRP3 licensing.

#### Phosphorylation and dephosphorylation

Accumulating evidence suggests that the control of phosphorylation/dephosphorylation of inflammasome components is required for the priming/licensing of NLRP3 inflammasome activation (Fig. 2). In the early phase of priming, c-Jun N-terminal kinase 1 (JNK1)-dependent phosphorylation of NLRP3 at human Ser198 (mouse Ser194) is critical for NLRP3 deubiquitination and self-association, which drive inflammasome activation.^55^ In addition, the NLRP3 inflammasome is phosphorylated at human Ser295 (mouse Ser293), and the role of this phosphorylation in NLRP3 activation is controversial. During priming, NLRP3 is phosphorylated at human Ser295 (mouse Ser293) by protein kinase D (PKD), an effector of diacylglycerol (DAG), at the Golgi apparatus, which is adjacent to mitochondria-associated ER membranes (MAMs), where NLRP3 and ASC assemble to form the inflammasome complex.^56^ However, another study showed that Ser295 phosphorylation by protein kinase A (PKA) has an inhibitory effect through suppression of the ATPase activity of the NLRP3 NACHT domain, which is critical for NLRP3 oligomerization.^57^ The molecular details of NLRP3 Ser295 phosphorylation are poorly understood. Further studies are required to explore the mechanisms underlying the dual functions involving the same phosphorylation site.

Interestingly, Bruton’s tyrosine kinase (Btk) may play dual opposite roles in the priming phase of NLRP3 inflammasome activation. A recent report showed that Btk promotes NLRP3 inflammasome activation through phosphorylation of ASC at Tyr144 and physical interaction with NLRP3 and ASC, thereby contributing to postischemic inflammation after stroke.^58^ However, another study reported that Btk interacts with NLRP3 during priming and functions as a physiological inhibitor of NLRP3 phosphorylation and oligomerization.^59^ The inhibitory function of Btk is mediated through the maintenance of NLRP3 phosphorylation at human Ser5 (mouse Ser3),^59^ which is in the PYD interaction interface.^9^ NLRP3 Ser5 phosphorylation is critical for suppression of NLRP3 inflammasome activation through interference with charge–charge interactions between PYD domains.^9^ Mechanistically, Btk suppresses protein phosphatase 2 A (PP2A), which dephosphorylates Ser5 of the PYD in NLRP3, thus blocking aberrant activation of the NLRP3 inflammasome and the related inflammation.^9,59^ These data may explain the observation that Btk-deficient macrophages or monocytes from patients with X-linked agammaglobulinemia (XLA) with Btk mutation have dysregulated NLRP3 inflammasome activity.^59^

Another transmembrane tyrosine kinase, EphA2, physically interacts with NLRP3 and induces its phosphorylation at Tyr132, thus inhibiting NLRP3 inflammasome assembly in murine airway epithelial cells during reovirus infection.^60^ In addition, EphA2-mediated NLRP3 phosphorylation is crucial for amelioration of pathological asthmatic exacerbation in a mouse model of asthma.^60^ The enhanced tyrosine phosphorylation of NLRP3 at Tyr861 negatively regulates inflammasome activation through activation of autophagy for NLRP3 degradation.^61^ Protein tyrosine phosphatase nonreceptor 22 (PTPN22) targets and dephosphorylates NLRP3 at tyrosine residue Tyr861, thereby activating the NLRP3 inflammasome and IL-1 secretion.^61,62^ Furthermore, phosphatase and tensin homolog (PTEN) in myeloid cells interacts with and dephosphorylates NLRP3 at Tyr32, thereby promoting assembly of the NLRP3 inflammasome.^63^ Given that PTEN-NLRP3 functions in enhancing chemotherapy sensitivity and antitumor responses,^63^ myeloid-specific NLRP3 regulation of phosphorylation may be associated with chemotherapeutic responsiveness in the tumor immune microenvironment.^63^ As apparent from these studies, NLRP3 activation by phosphorylation/dephosphorylation is regulated in a multilayered manner. Future studies are warranted to clarify how multiple tyrosine kinases and phosphatases orchestrate the fine-tuning of NLRP3 inflammasome activation and their functional consequences in a variety of human diseases.

#### Other PTMs: acetylation/deacetylation, SUMOylation, and nitrosylation

Several types of PTMs, including acetylation/deacetylation, SUMOylation, and nitrosylation, are also involved in the regulation of NLRP3 inflammasome activation (Fig. 2). Previous studies showed that nitric oxide (NO) and S-nitrosylation of NLRP3 inhibit inflammasome assembly and IL-1 production during mycobacterial infection and LPS stimulation.^64–66^ However, whether nitrosylation is required for NLRP3 priming or feedback regulation after NLRP3 inflammasome activation remains to be fully characterized. In addition, NLRP3 SUMOylation plays either a positive or negative role in NLRP3 inflammasome activation depending on the context. NLRP3 SUMOylation by the small ubiquitin-like modifier (SUMO) E3 ligase MAPL (MUL1) restrains activation of the NLRP3 inflammasome,^67^ suggesting that SUMO conjugation of NLRP3 at multiple sites is a fundamental negative regulator of innate immune signaling. However, another study showed that SUMOylation of NLRP3 at K204 by SUMO1 facilitates ASC oligomerization and NLRP3 inflammasome activation.^68^ Additional studies are needed to understand how multiple PTM pathways are selected and coordinated for the priming/licensing of NLRP3 and its oligomerization.

A recent study showed that SIRT2-mediated deacetylation of NLRP3 ameliorates NLRP3 inflammasome activation, thus contributing to protection against aging-associated inflammation and insulin resistance.^69^ However, it is unclear whether multiple Lys residues of NLRP3 are acetylated or deacetylated under basal conditions and which upstream signals regulate the acetylation of NLRP3 at certain phases of inflammasome activation. In addition, future studies should investigate whether a variety of PTMs play synergistic or redundant roles in NLRP3 priming/licensing. During NLRP3 inflammasome activation, various types of PTMs, including phosphorylation, ubiquitination, and SUMOylation, might be activated sequentially or simultaneously in a context-specific manner. Whether different types of PTMs are activated in an interlinked, overlapping, or independent manner remains a major theme to be explored in terms of the NLRP3 inflammasome licensing step.

#### NLRP3 interactions with molecular partners

##### NLRP3 and NEK7 interaction

The mitotic serine and threonine kinase NEK7, a member of the mammalian never in mitosis A (NIMA)-related kinase (NEK) protein family, is a key interacting partner of NLRP3, leading to NLRP3 oligomerization along with ASC speck formation and maturation of IL-1β and IL-18 in response to NLRP3 inflammasome activating signals involving K^+^ efflux and ROS.^70–72^ NEK7 is also transcriptionally activated by RELA through direct targeting and activation of NLRP3 promoter activity.^73^ There are two major isoforms of human NLRP3 produced by alternative splicing, i.e., the full-length variant and a variant that lacks exon 5 and cannot interact with NEK7, resulting in the attenuation of NLRP3 inflammasome activation.^74^ A recent structural modeling study using cryoelectron microscopy highlighted the molecular mechanism of NEK7–NLRP3 interactions—NEK7 was shown to bridge adjacent NLRP3 subunits and facilitate NLRP3 inflammasome oligomerization.^10^

Whether NEK7 is absolutely required for NLRP3 oligomerization and further facilitation of inflammasome assembly remains an unknown. A recent preprint suggested NEK7-independent but TGF-β-activated kinase-1 (TAK1)-dependent PTM regulation of NLRP3 priming.^75^ Further understanding of NEK7-dependent and NEK7-independent priming pathways and how they work together or separately will provide more precise insights into the molecular mechanisms involved in licensing NLRP3 inflammasome activation.

##### NLRP3 interactions with other proteins

Studies showed have shown that thioredoxin-interacting protein (TXNIP) binds to NLRP3 in a redox-dependent manner and plays a critical role in the activation of the NLRP3 inflammasome.^76–78^ In addition, mitochondrial antiviral signaling (MAVS) protein plays a key role in the optimal activity of the NLRP3 inflammasome through binding with and recruitment of NLRP3 in response to RNA viruses and synthetic RNA polyinosinic–polycytidylic acid.^79–81^ During infection with RNA viruses, including influenza virus and encephalomyocarditis virus, the outer mitochondrial membrane protein mitofusin 2 (MFN2) physically interacts with NLRP3 and further induces the secretion of IL-1β through NLRP3 inflammasome activation.^82^

During cellular stress responses, stress granules are critical to cell survival. A recent study showed that the stress granule protein DEAD-box helicase 3 X-linked (DDX3X) interacts with NLRP3 to promote inflammasome activation. The assembly of stress granules and NLRP3 inflammasome pathways compete for DDX3X in innate immune responses and the determination of cell fate under stress conditions.^83^ In NLRP3 inflammasome activation, the stress granule protein DDX3X interacts with NLRP3, leading to the formation of pyroptotic ASC specks.^83^ The rheostat-like function of DDX3X between activation of the NLRP3 inflammasome and stress granule assembly may contribute to the determination of live-or-die cell fate decisions in response to stressors.^83^

More recent studies have shown that microtubule affinity regulating kinase 4 (MARK4) physically interacts with NLRP3 and drives its localization to the microtubule-organizing center, contributing to the formation of the NLRP3 inflammasome complex.^84^ It was also reported that E74-like ETS transcription factor 3 (ELF3) increases MARK4 expression upon high glucose-triggered NLRP3 inflammasome activation in vascular endothelial cells.^85^ In addition, there are several other molecular partners (ZBP1, caspase-8/FADD, etc.) that interact with NLRP3 in the context of pyroptosis or priming signaling. These molecules have been discussed in the relevant section in this review. Further studies are warranted to clarify which protein partners are recruited to NLRP3 to activate the inflammasome complex further and how each can be regulated in the respective context.

#### Spatiotemporal activation of the NLRP3 inflammasome complex

##### MAMs and the microtubule-organizing center (MTOC)

**Fig. 3:**
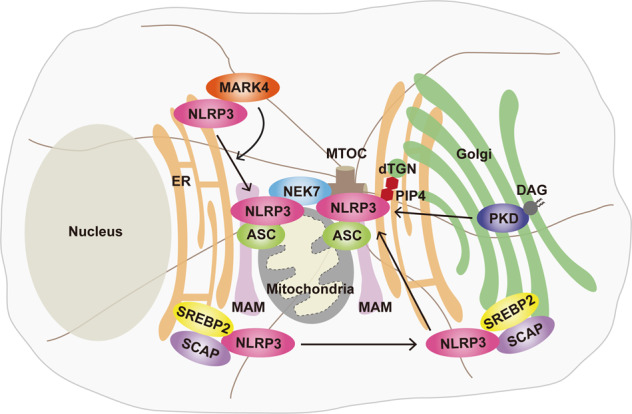
Spatiotemporal regulation of the NLRP3 inflammasome complex. NLRP3, which is present in the endoplasmic reticulum (ER), comes in close proximity to apoptosis-associated speck-like protein containing a CARD (ASC) in mitochondria upon induction by various stimuli. It is known that NLRP3 and ASC are assembled in mitochondria-associated ER membranes (MAMs). NLRP3 binds to microtubule affinity regulating kinase 4 (MARK4) and is translocated to microtubule-organizing center (MTOC). When NLRP3 reaches the MTOC, NIMA related kinase 7 (NEK7) binds with NLRP3, and the inflammasome is assembled. In addition, NLRP3 is recruited to the dispersed *trans*-Golgi network (dTGN) through phosphatidylinositol-4-phosphate (PIP4) by diverse stimuli. In addition, translocation of the SREBP cleavage-activating protein (SCAP)-sterol regulatory element-binding protein 2 (SREBP2) complex from the ER to the Golgi is important for the activation of NLRP3. Diacylglycerol (DAG) in the Golgi recruits protein kinase D (PKD), which is involved in the assembly and activation of NLRP3

NLRP3 resides in the endoplasmic reticulum (ER) and cytosol, and upon activation by diverse stimuli, NLRP3 in the ER localizes adjacent to ASCs in mitochondria (Fig. 3).^86,87^ Indeed, the NLRP3 inflammasome complex can be assembled at highly specialized contact sites between the ER and mitochondria known as MAMs.^86^ Mitochondrial ASC apposition to ER NLRP3 is mediated through acetylated α-tubulin via dynein-dependent mitochondrial transport to the ER.^87^ The localization of NLRP3 to MAMs/mitochondria may contribute to the immediate recognition of and response to mitochondrial damage, mitochondrial DNA (mtDNA) translocation, and cardiolipin.^86^

Several recent studies have revealed the molecular mechanisms by which microtubules provide the optimal sites for the activation of the NLRP3 inflammasome. The binding between MARK4 and NLRP3 results in the translocation of NLRP3 into the MTOC, where inflammasome speck formation and assembly are activated.^84^ Importantly, the localization of NLRP3 to the MTOC leads to its interaction with the centrosome-localized mitotic kinase NEK7 to facilitate NLRP3 inflammasome assembly.^10^ Furthermore, a recent study showed that the dynein adapter histone deacetylase 6 (HDAC6) is critical for microtubule transport, inflammasome assembly, and autophagosomal degradation of aggresomes at the MTOC, the centrosome.^88^ Studies are beginning to reveal where when, and how the NLRP3 inflammasome complex is assembled, depending on the context. A deeper understanding of the mechanisms underlying the proximity to several subcellular compartments will contribute to the identification of potential therapeutic targets for NLRP3-related disorders.

##### Trans-Golgi disassembly

Recent studies have highlighted that the Golgi apparatus and its lipid mediators play essential roles in the aggregation of NLRP3 and the activation of NLRP3 inflammasome assembly.^88–90^ Imaging and biochemical analyses showed that NLRP3 exposed to certain stimuli induces the disassembly of the *trans*-Golgi network (TGN) into the dispersed TGN (dTGN) and that NLRP3 is recruited to the dTGN via the conserved polybasic region of NLRP3. Indeed, the phospholipid phosphatidylinositol-4-phosphate (PIP4) is exposed on dTGN and recruits and interacts with NLRP3, thus resulting in the formation of multiple NLRP3 puncta and caspase-1 activation. Notably, K^+^ efflux-independent stimuli (imiquimod) the high activation of NLRP3-dTGN, which leads to aggregation and activation of the NLRP3 inflammasome.^89^ In addition, NLRP3 inflammasome activation is dependent on the ER-to-Golgi translocation of sterol regulatory element-binding protein (SREBP) 2 and SREBP cleavage-activating protein (SCAP), which form a ternary complex with NLRP3.^90^ How then does NLRP3 inflammasome assembly occur at the intracellular level in both MAMs and dTGN? Another recent study reconciled this issue by demonstrating that NLRP3 inflammasome stimuli induce the localization of MAMs adjacent to Golgi membranes.^56^ This interorganelle communication depends on the recruitment of PKD to the sites of DAG at the Golgi, thereby facilitating NLRP3 oligomerization and assembly of the active inflammasome.^56^

Taken together, these data suggest that the spatial interrelations among the ER-mitochondria-Golgi apparatus are closely related to NLRP3 inflammasome activation (Fig. 3). Given that K^+^ efflux-dependent and K^+^ efflux-independent stimuli converge for Golgi disassembly,^89^ future studies should consider simultaneous measurement of NLRP3–ASC aggregation in different organelles and develop inclusive approaches that address not only signaling and cytokine production but also organellar contacts and tethering to exert crucial control over spatiotemporal activation of the NLRP3 inflammasome. Moreover, recent findings suggest that ASC specks can be secreted or found ex vivo.^91,92^ Further studies are needed to clarify how spatiotemporal coordination among intracellular organelles regulates the ASC secretion pathway and to decipher the complex interrelationships between ASC and other inflammasome components that affect the distinct physiological and pathological roles of secreted ASC in terms of NLRP3 inflammasome activation.

#### Signal 2: activation of the NLRP3 inflammasome

A variety of stimuli that perturb intracellular ion homeostasis, i.e., K^+^ efflux, intracellular Ca^2+^ flux, and Cl^−^ efflux, can activate the assembly of the NLRP3 inflammasome complex and release mature IL-1β. Other pathways, including mitochondrial dysfunction, lysosomal destabilization, and metabolic alteration pathways, also contribute to NLRP3 inflammasome activation. Recent studies have indicated that several other pathways, including the complement, protein kinase R (PKR), purine receptor signaling, necroptosis, and ZBP1 pathways, are required for Signal 2 of NLRP3 inflammasome activation. In this section, we discuss recent advances in the understanding of the pathways and mechanisms by which Signal 2 triggers activation of the NLRP3 inflammasome complex.

##### Potassium efflux

K^+^ efflux has emerged as a common step in the activation of the NLRP3 inflammasome induced by multiple NLRP3 agonists, including nigericin, a well-known K^+^/H^+^ ionophore, and extracellular ATP.^72,93–95^ The channel-forming glycoprotein pannexin-1 hemichannels are known to be involved in inflammasome activation through membrane permeability and ATP release during apoptosis.^96,97^ It was thought that ATP gating of the P2X7 receptor (P2X7R), an ion channel in the purinergic receptor family, promotes IL-1β maturation via K^+^ efflux.^95,98^ A recent study showed that tandem pore domains in weak inward rectifying K^+^ channel 2 (TWIK2) mediate K^+^ efflux in cooperation with P2X7R-mediated influx of Ca^2+^ and Na^+^, leading to ATP-mediated activation of the NLRP3 inflammasome in macrophages.^99^ Notably, neither nigericin- nor imiquimod-induced NLRP3 inflammasome activation was regulated in TWIK2-deficient macrophages.^99^

Indeed, numerous signals, including the complement cascade component membrane attack complex (MAC)^100,101^ and particulate matter signals,^95^ converge for the induction of K^+^ efflux to promote NLRP3 inflammasome activation. In addition, *Mycobacterium tuberculosis* (Mtb) infection results in ESX-1-mediated plasma membrane damage responses that cause K^+^ efflux, leading to the activation of caspase-1/NLRP3/GSDMD-mediated pyroptosis in human monocytes and macrophages.^102^ Phospholipids, platelet-activating factor (PAF), and PAF-like lipids can activate the canonical NLRP3 inflammasome through mechanisms involving K^+^ efflux and Ca^2+^ influx in a manner independent of the PAF receptor.^103^ Although K^+^ efflux is required for the NEK7-NLRP3 association,^72^ the mechanisms underlying K^+^ efflux-mediated NLRP3 inflammasome activation are not fully understood. In this regard, further studies are warranted to elucidate how K^+^ efflux triggered by multiple signals activates the assembly of the NLRP3 inflammasome complex.

##### Calcium signaling and calcium-sensing receptor (CaSR)

Intracellular Ca^2+^ flux plays an essential role in the assembly and activation of the NLRP3 inflammasome induced by multiple stimuli.^104,105^ Studies showed that CaSR signaling promotes NLRP3 inflammasome assembly through intracellular Ca^2+^ flux and leads to a decrease in the cellular level of cyclic AMP (cAMP), which is an inhibitory signal for inflammasome activation.^105^ Recent studies have shown that platelets are able to boost NLRP3 inflammasome activation by triggering CaSRs in human macrophages, suggesting the importance of Ca^2+^ signaling in the activation of the inflammasome linked to cell–cell interactions.^106^ Indeed, intracellular Ca^2+^ mobilization seems to be involves in coordinated action with several other signaling pathways to activate the NLRP3 inflammasome complex. Several studies have shown a cooperative relationship between K^+^ efflux and Ca^2+^ flux, contributing to the idea of a greater influence on mtROS generation.^99,107^ However, other studies have shown that K^+^ efflux-mediated NLRP3 inflammasome signaling is not associated with cytosolic Ca^2+^ flux.^108,109^ In addition, Ca^2+^ flux-mediated calpain activation is required for caspase-1 activation, whereas K^+^ efflux inhibits calpain.^109^ Therefore, the cross talk between Ca^2+^ flux and K^+^ efflux pathway components is complex, regulated in a context-dependent manner, and remains to be fully elucidated.

Recent studies have revealed relationships of Ca^2+^ flux and oxidative stress in the activation of the NLRP3 inflammasome. Particulate matter-mediated oxidative stress can trigger activation of the NLRP3 inflammasome through intracellular Ca^2+^ mobilization.^110^ In this case, transient receptor potential melastatin 2 (TRPM2), a calcium-permeable cation channel, mediates ROS-associated NLRP3 inflammasome activation.^110,111^ Apolipoprotein C3 (ApoC3)-triggered alternative NLRP3 inflammasome activation involves intracellular Ca^2+^ flux and the production of ROS in human monocytes.^112^

Ca^2+^ flux-triggered NLRP3 inflammasome activation is closely associated with the pathogenesis of several human autoimmune diseases. Extracellular ADP, a danger signal, is extensively released from injured colonic tissue in inflammatory bowel disease. ADP/P2Y_1_ receptor signaling activates the NLRP3 inflammasome through intracellular Ca^2+^ mobilization, thereby aggravating intestinal inflammation.^113^ In addition, the increased extracellular Ca^2+^ and phosphate induced by the formation of fetuin-A-based calciprotein particles triggers NLRP3 inflammasome activation through CaSR-mediated signaling, leading to pathological inflammation in inflammatory arthritis.^114^ Taken together, these data suggest that Ca^2+^ flux-induced signaling depends on another molecule/pathway to integrate sufficient signals for NLRP3 inflammasome activation. It will be important to explore further the molecular mechanisms by which signals selectively and cooperatively impact the ability of Ca^2+^ flux to activate the NLRP3 inflammasome.

#### Chloride efflux

The decreased extracellular Cl^−^ level, which often acts in cooperation with other signals for NLRP3 activation, promotes activation of caspase-1, leading to mature IL-1β secretion.^12,115^ During inflammasome activation, the chloride intracellular channel (CLIC) proteins CLIC1 and CLIC4 are translocated to the plasma membrane where they mediate Cl^−^ efflux.^116^ In addition, CLICs function as proximal and upstream signals for priming by synthesizing IL-1β and as downstream signals of the K^+^ efflux–mtROS axis for NLRP3 inflammasome activation.^115,116^ However, another report indicated that K^+^ and Cl^−^ efflux is required for the oligomerization of NLRP3 and ASC, respectively,^117^ suggesting that both K^+^ and Cl^−^ efflux pathways function separately in the activation of the NLRP3 inflammasome.

Cystic fibrosis is caused by genetic mutations of cystic fibrosis transmembrane conductance regulator (CFTR), which is an ion channel involved in the transport of chloride and bicarbonate with hyperabsorption of sodium due to a dysregulated epithelial sodium channel (ENaC).^118,119^ Although the mechanisms underlying excessive inflammation in patients with cystic fibrosis remain a matter of some debate,^118^ it is suggested that ENaC-mediated Na^+^ influx, accompanied by defective Cl^−^ efflux, may contribute to exaggerated inflammatory responses and NLRP3 inflammasome activation in this disease.^120^ Given the role of Cl^−^ efflux in NLRP3 inflammasome induction, a recent study revealed a new mechanism of action of the FDA-approved drug ticagrelor, which is used for the treatment of coronary artery disease.^121^ Ticagrelor functions by attenuating the oligomerization of ASCs by blocking Cl^−^ efflux via the degradation of CLICs and inhibition of their translocation to the plasma membrane.^121^ However, another recent study showed that myocardial protection by ticagrelor is mediated through its antiplatelet properties and not an additive effect involving the inhibition of the NLRP3 inflammasome.^122^ Understanding the mechanisms by which Cl^−^ efflux controls NLRP3 inflammasome activation may facilitate the discovery of novel agents or drugs suitable for repurposing to increase clinical benefit for patients with one of a variety of NLRP3-related diseases.

##### Mitochondrial dysfunction, oxidative stress, mtDNA, and mitochondrial dynamics

Beyond their role in energy metabolism, mitochondria are emerging as central organelles in the activation of the NLRP3 inflammasome. Mitochondria can play multifaceted roles by serving as docking sites for assembly of the NLRP3 inflammasome, release of danger signals, generation of mtROS, etc.^12,123–125^ Persistent damage and dysfunction of mitochondria, often induced by a wide range of danger signals, are key upstream processes for activation of the NLRP3 inflammasome.^1,12,126–128^ Mitochondrial dysfunction provides the key activation mechanism for the NLRP3 inflammasome complex through excessive generation of mtROS, cytosolic translocation of mtDNA, or relocation of mitochondria to the proximity of NLRP3 by the induction of α-tubulin acetylation.^12,17,128–130^ Increased mitochondrial stress often lead to detrimental consequences that contribute to the pathogenesis of metabolic diseases.^128,130^ Although the supposition remains controversial, mitochondrial dysfunction has been suggested to be closely linked with other signaling pathways, including K^+^ efflux or Cl^−^ efflux pathways, for activating the NLRP3 inflammasome.^12,17,116,128,130^

Several small molecules that target mitochondria lead to the production of mtROS to further activate the NLRP3 inflammasome complex. For example, imiquimod, a small-molecule ligand activates TLR7, and the related compound, CL097, activates the NLRP3 inflammasome through the production of mtROS, but K^+^ efflux is not involved.^131^ In addition, oxidation of phosphatidylcholine upon cellular stress and damage activates the NLRP3 inflammasome in macrophages through mtROS downstream of intracellular Ca^2+^ signaling.^132^ A recent study of neuroinflammation in a model of Parkinson’s disease showed that the Fyn kinase induces PKCδ-dependent NF-κB-p65 activation and inflammasome priming. This activation and priming facilitate α-synuclein uptake by microglia, contributing to the generation of mtROS and leading to exaggerated neuroinflammation and progression of Parkinson’s disease.^133^ Human respiratory syncytial virus (RSV) infection triggers macrophage cell lysis through NLRP3 inflammasome-mediated pyroptosis through ROS production^134^ Moreover, supraphysiological testosterone levels trigger vascular dysfunction through induction of mtROS generation, enhancing NLRP3 inflammasome activation and leading to increased cardiovascular risk.^135^ In summary, multiple danger or microbial signals are involved in triggering mtROS generation to further activate the NLRP3 inflammasome.

Indeed, NLRP3 signaling activators result in mitochondrial destabilization and the release of mitochondria-derived molecules, such as mtDNA and cardiolipin, to further activate the NLRP3 inflammasome complex.^124,130,136^ Circulating mitochondrial DAMPs, including formyl peptides and mtDNA, can be produced upon cellular injury, and they induce systemic inflammation.^137^ More recently, it was shown that mtDNA synthesis following TLR signaling can lead to the formation of oxidized mtDNA fragments that lead to inflammasome activation, indicating that these fragments are critical components of the NLRP3 machinery.^138,139^ Severe fever with thrombocytopenia syndrome (SFTS) virus (SFTSV) infection triggers the activation of BCL2 antagonist/killer 1 (BAK)/BCL2-associated X (BAX) signaling, leading to mitochondrial dysfunction and release of oxidized mtDNA that activates the NLRP3 inflammasome.^140^ Furthermore, the mitochondrial phospholipid cardiolipin appears to be a signaling platform for autophagy, apoptosis, and inflammasome activation.^141^ Cardiolipin binds directly to the LRR domain of NLRP3 and provides an activating signal for NLRP3 inflammasome complex assembly and activation.^123,125^ A deeper, context-dependent understanding of the roles of mtROS and mtDNA and the interaction of NLRP3 components with mitochondrial molecules is required to appreciate inflammasome formation and understand the pathophysiological effects of the inflammasome.

Mitochondrial dynamic proteins may play key roles in inflammasome activation, which is related to chronic inflammation in type 2 diabetes mellitus (T2DM).^142^ Under conditions of nutrient excess, the expression of inflammasome-related genes and inflammatory responses are increased in cybrid cells harboring mitochondrial haplogroup B4, which is the type 2 diabetes-associated haplogroup in the Chinese population. Notably, inflammasome-related inflammatory responses are attenuated by inhibition of Drp1 and overexpression of fusion proteins, suggesting that inflammasome activation is regulated by components involved in mitochondrial dynamics.^142^ However, as mentioned above, MFN2 interacts with NLRP3 and activates the inflammasome during RNA virus infection.^82^ Thus, the issues of mitochondrial dynamics and inflammasome activation remain to be addressed before we can gain a deeper understanding of the diverse effects of each component in mitochondrial dynamics on inflammasome regulation.

##### Lysosomal disruption

Studies showed that particulate matter, including uric acid and cholesterol crystals, alum, silica, and asbestos, are canonical stimulators of NLRP3 inflammasome activation through induction of lysosomal damage and rupture, thereby releasing multiple cathepsins into the cytoplasm.^143,144^ Recent studies have shown that carbon-based nanomaterials, such as multiwalled carbon nanotubes, can activate the NLRP3 inflammasome through lysosomal destabilization and release of cathepsin B.^145^ Nicotine also induces lysosomal membrane permeability in endothelial cells and triggers the lysosomal release of cathepsin B, thus enhancing NLRP3 inflammasome activation.^146^ Recent studies have suggested a more generalized function of cathepsin B in the activation of the NLRP3 inflammasome through a direct interaction with NLRP3 at the ER upon stimulation with multiple types of NLRP3 activators, including ATP and nigericin, as well as particulate matter.^147^

Although the interwoven molecular pathways are not well understood, lysosomal damage and rupture may require another signal for full activation of the NLRP3 inflammasome. For example, Leu-Leu-O-methyl ester (LLME), a soluble lysosomotropic agent, induces NLRP3 inflammasome activation through the combined effects of lysosome membrane permeabilization and increased K^+^ efflux.^148^ In addition, lysophosphatidylcholine (LPC), a major lipid component in the plasma membrane, activates foam cell formation and triggers NLRP3 inflammasome activation in human endothelial cells and monocytes upon lysosomal damage and K^+^ efflux.^149^ Recent studies have also shown that plasma membrane damage is a key upstream event for lysosomal damage-associated NLRP3 inflammasome activation.^102^ During *Candida albicans* infection, the expansion of phagosomes through lysosome recruitment is needed to prevent NLRP3 inflammasome activation and host cell death.^150^ However, phagosomal rupture and/or lysosomal damage triggers NLRP3 inflammasome activation at least partly through plasma membrane damage.^102,150^ Future studies should examine the detailed molecular mechanisms underlying the cross talk between molecules involved in plasma damage and lysosomal destabilization-associated NLRP3 inflammasome activation and pyroptosis.

##### Complement system and PKR pathway

There is accumulating evidence indicating that a variety of elements in innate immune responses are essential activators of the NLRP3 inflammasome. For example, the complement system is essentially involved in the activation of inflammasome pathways in the context of autoimmune and inflammatory responses. As mentioned above, the complement cascade component MAC can trigger NLRP3 inflammasome activation and pathological inflammation.^100,101^ Ischemia-reperfusion injury (IRI) results in immunoglobulin M (IgM)-dependent complement system activation that induces NLRP3 inflammasome assembly in endothelial cells.^151^ The internalization of MAC in IFN-γ-primed human endothelial cells causes NLRP3 translocation into endosomes and leads to endosomal NF-κB-inducing kinase (NIK)-dependent inflammasome assembly, resulting in complement-associated pathologies.^152^ Several studies have revealed that the C5a/C5aR pathway promotes activation of the NLRP3 inflammasome through amplification of dsRNA-dependent PKR expression in macrophages, suggesting that PKR is an important NLRP3-activating factor.^153^ In addition, the C5a/C5aR2 axis-dependent induction of HMGB1 contributes to pathological damage and renal inflammation through upregulation of NLRP3 inflammasome activation in macrophages.^154^ Taken together, these studies suggest a molecular link is established between the complement system and the NLRP3 inflammasome in a multilayered and complex way to potentiate inflammatory pathology in a variety of NLRP3-associated disorders. Further studies are needed to determine the precise mechanisms underlying the interrelationship between complement system components, PKR, and inflammasome activation.

##### Purine receptor signaling

Adenosine and ATP receptors are involved in a variety of metabolic and degenerative diseases through inflammasome activation.^155^ P2X7R, a distinct ligand-gated ion channel, is recognized as a strong activating signal for NLRP3 inflammasome assembly and secretion of IL-1β. The P2X7R-cathepsin pathway contributes to pathological inflammation in a variety of autoimmune diseases, including systemic lupus erythematosus (SLE), rheumatoid arthritis (RA), and inflammatory bowel disease (IBD).^98^ P2Y_14_ receptor (P2Y_14_R) participates in the induction of caspase-1-mediated pyroptosis through inhibition of adenylyl cyclase and suppression of cAMP/NLRP3 signaling, thereby contributing to exacerbation of inflammation in acute gouty arthritis and pyroptosis-related diseases.^156^ In addition, extracellular ADP triggers NF-κB signaling and NLRP3 inflammasome activation to enhance microglial inflammation through the P2Y_12_ receptor, a metabotropic P2YR expressed in microglia.^157^ Collectively, these findings warrant a more comprehensive assessment based on purine receptor signaling-mediated inflammasome modulation to explore their clinical therapeutic efficacy in various NLRP3-associated diseases.

##### Necroptotic signaling and ZBP1

Emerging data suggest a close relation between the NLRP3 inflammasome and RIPK1/3-mediated necroptosis pathways. Necroptotic signaling mediated by RIPK1, RIPK3, and MLKL activates the NLRP3 inflammasome to enhance IL-1β, suggesting that this cell death pathway is closely associated with NLRP3 inflammasome activation and the pathogenesis of heritable autoinflammatory diseases.^158–160^ RIPK1 kinase activity is generally related to PANoptosis (pyroptosis, apoptosis, and necroptosis). In TAK1-deficient macrophages, autocrine TNF signaling, without TLR priming, induces spontaneous RIPK1-dependent NLRP3 inflammasome activation and cell death.^161^ Orning et al. also reported RIPK1- and caspase-8–dependent cleavage of GSDMD, resulting in pyroptosis and the release of IL-1β and IL-18 by inhibiting TAK1–IκB kinase signaling with the *Yersinia* effector protein YopJ.^162^ Interestingly, TLR priming that mimics pathogen-mediated priming triggers RIPK1 kinase activity-independent PANoptosis and activation of the NLRP3 inflammasome in the absence of TAK1.^163^ Moreover, TAK1 inactivation leads to myeloid proliferation and severe systemic inflammation through the RIPK3-caspase-8 signaling axis in vivo.^163^ These results support the supposition that TLR priming during TAK1 deficiency bypasses the RIPK1 requirement, but not RIPK3 and caspase-8, which are needed to induce pyroptotic cell death and inflammation in macrophages. We are only beginning to understand the complex regulatory mechanisms between innate immunity, inflammatory cell death, and NLRP3 inflammasome activation. Further studies are needed to identify the potential factors and mechanisms to explain how the sum of these pathways determines the pathophysiological consequences during numerous inflammatory and infectious diseases.

Recent studies have indicated the impact of regulators of necroptosis on NLRP3 activation-related pathologies. For example, sirtuin 3, a major deacetylase involved in mitochondrial homeostasis, is required to control the expression of necroptosis-related RIPK1, RIPK3, and NLRP3, as well as to prevent mitochondrial injury and mtROS, thereby exerting a protective effect in diabetic cardiomyopathy.^164^ In addition, the RIPK3 inhibitor dabrafenib was shown to be beneficial for amelioration of renal fibrosis, the pathogenesis of which is associated with RIPK3-regulated NLRP3 inflammasome activation.^165^

As mentioned above, ZBP1 represents the key mediator of NLRP3 inflammasome-related cell death. During influenza virus infection, an innate immune sensor and the interferon-inducible protein ZBP1 can sense Z-RNA and trigger cell death through PANoptosis (pyroptosis, apoptosis, and necroptosis) through the multiprotein PANoptosome complex via formation of the ZBP1-NLRP3 inflammasome.^24,166–168^ The ZBP1 Zα2 domain is crucial for influenza A virus (IAV)-induced PANoptosis, NLRP3 inflammasome activation, and perinatal lethality, which are associated with hyperinflammation and epithelial damage.^166,169^ In addition, caspase-6 is required for ZBP1-mediated inflammasome activation by facilitating the binding of RIPK3 to ZBP1.^170^ Furthermore, IFN regulatory factor (IRF)1 is a transcriptional regulator of ZBP1 and promotes activation of the NLRP3 inflammasome and induces cell death during IAV infection.^171^ Further studies to elucidate the cellular and molecular mechanisms underlying ZBP1-mediated NLRP3 inflammasome activation and PANoptosis may enable the identification of new therapeutic agents useful for the termination of severe viral infections and the design of novel vaccines.

#### Dual regulatory mechanisms controlling the NLRP3 inflammasome

##### Immunometabolism (positive regulation)

The metabolic reprogramming of immune cells plays a critical role in the regulation of inflammatory responses and NLRP3 inflammasome activation (Table 1).^20,172^ Enhanced glycolysis coupled with increased succinate levels increases IL-1β expression by stabilizing HIF-1α in macrophages.^173^ Glycolysis-related activation of mitochondrial respiration and an increase in mtROS levels contribute to activation of the NLRP3 inflammasome and IL-1β secretion.^174^ In addition, pyruvate kinase isozyme M2 (PKM2)-mediated aerobic glycolysis drives inflammasome activation through phosphorylation of eukaryotic translation initiation factor 2-alpha kinase 2 (EIF2AK2)/PKR in macrophages^175^ In postburn responses with abnormal scar formation (keloid), NLRP3 inflammasome activation is correlated with glucose transporter 1 (GLUT1) expression and glycolysis. The inhibition of aberrant glucose metabolism attenuates NLRP3 inflammasome activation, suggesting that Warburg-like metabolism is closely associated with NLRP3-mediated inflammation in postburn responses.^176^

**Table 1:** Dual regulatory mechanism of immunometabolism in controlling inflammasome

Regulator	Mechanism	Study model	Ref.
Immunometabolism (Positive Regulation)			
Glucose metabolism			
Succinate	Upregulation of IL-1β expression by stabilizing HIF-1α in macrophages	BMDMs, C57BL/6 mice	^173^
PKM2	PKM2-mediated aerobic glycolysis by phosphorylation of EIF2AK2	Mouse PMs, BMDMs, BALB/c mice	^175^
	Enhanced glycolysis with elevated PKM2 and GLUT1 expression	Keloid tissue from human patients, C57BL/6 mice	^176^
N-acetylglucosamine	Inhibition of hexokinase and induction of its dissociation from mitochondrial outer membrane	BMDMs, dendritic cells, C57BL/6 mice	^177^
Glucose starvation	Metabolic competition by *C. albicans* triggering glucose starvation	BMDMs, human MDMs	^178^
Lipid metabolism			
Cholesterol	NPC1-dependent cholesterol efflux from late endosome-lysosome compartment to ER	primary and immortalized BMDMs	^180^
	ER-to-Golgi translocation and complex formation of SCAP-SREBP2 with NLRP3	HEK293T and THP-1 cells, BMDMs, C57BL/6 mice	^90^
PIP4	Binding of NLRP3 to the dispersed TGN	HeLa, COS-7, and RAW264.7 cells, BMDMs	^89^
Immunometabolism and immune reprogramming (Negative Regulation)			
Krebs cycle			
4-octyl itaconate	Blockage of NLRP3-NEK7 interaction through the modification of C548 on NLRP3	BMDMs, human PBMCs, C57BL/6 mice	^183^
Ketone bodies			
BHB	SGLT2 inhibitor-mediated reduction of IL-1β secretion with increased serum BHB and decreased serum insulin	T2DM patients with high CV risk, human macrophages	^184^
	Inhibition of K^+^ efflux and reduction of ACS oligomerization and speck formation	BMDMs, human monocytes, C57BL/6 mice	^185^
Glycolysis			
Cbl	Phosphorylation at Tyr371 and reduction of phosphorylated Pyk2 and mtROS level	BMDMs, THP-1 cells, C57BL/6 mice	^187^
Inhibition of GLUT1-dependent glycolysis	THP-1 cells	174	
Polysaccharide			
β-glucan	Inhibition of ASC oligomerization and speck formation through supression of K + efflux and mtROS generation	Human PBMCs, patients with CAPS	^188^

*BMDMs* bone marrow-derived macrophages, *PKM2* pyruvate kinase isozyme M2, *EIF2AK2* eukaryotic translation initiation factor 2-alpha kinase 2, *PM* peritoneal macrophages, *GLUT1* glucose transporter 1, *MDM* monocyte-derived macrophages, *NPC-1* Niemann-Pick C1, *SCAP* SREBP cleavage-activating protein, *SREBP2* sterol regulatory element-binding protein 2, *PIP4* phosphatidylinositol-4-phosphate, *TGN* trans-Golgi network, *PBMCs* peripheral blood mononuclear cells, *BHB* β-hydroxybutyrate, *SGLT2* sodium-glucose cotransporter 2, *T2DM* type 2 diabetes mellitus, *CAPS* cryopyrin-associated periodic syndrome

However, a recent study showed that during bacterial infection, N-acetylglucosamine, a sugar subunit of bacterial cell wall peptidoglycan, can inhibit and drive the relocalization of the glycolytic enzyme hexokinase from mitochondria into the cytosol. This localization leads to NLRP3 inflammasome activation that is independent of K^+^ efflux or pyroptosis.^177^ Inhibition of hexokinase, glycolytic inhibitors, and hexokinase relocalization appear to be sufficient to induce inflammasome activation.^177^ It remains to be determined how hexokinase in the cytosol triggers NLRP3 inflammasome assembly and activation. Although the mechanisms are not clear, competition of *C. albicans* with host cells for the use of glucose triggers activation of the NLRP3 inflammasome under conditions of glucose starvation caused by increased bacterial load.^178^ Accumulating data support future directions for study, such as investigating how immunometabolic regulation in the context of host–pathogen interactions shapes the collective outcome of infectious diseases.

As danger signals, cholesterol crystals trigger NLRP3 inflammasome activation, and dysregulated lipid metabolism plays a critical role in inflammasome-related diseases.^179^ The cholesterol trafficking pathway, the lysosomal efflux of cholesterol through Niemann-Pick C1 (NPC1), is tightly associated with immune responses, particularly NLRP3 inflammasome activation.^180^ In addition, the interaction of the cholesterol homeostatic regulator SCAP-SREBP2 with NLRP3 to form a complex that is translocated to the Golgi apparatus leads to the activation of the NLRP3 inflammasome in macrophages.^90^ Indeed, several lipids, including PIP4, contribute to NLRP3 aggregation and activation of the inflammasome complex.^89,179^ These data suggest that metabolic enzymes and metabolite changes directly activate the NLRP3 inflammasome complex. It is likely that data on the detailed molecular mechanisms underlying the regulation of immunometabolism will continue to accumulate in the context of inflammasome activation.

##### Immunometabolism and immune reprogramming (negative regulation)

Depending on which metabolites or metabolic pathways are predominant in individual immune cells in response to external cues, immunometabolic remodeling mechanisms can act as checkpoints to inhibit NLRP3 inflammasome activation.^20^ The important immunometabolite itaconate attenuates LPS-induced IL-1β secretion by impairing glycolytic flux by targeting the glycolytic enzymes fructose-bisphosphate aldolase A and GAPDH to enhance anti-inflammatory responses.^181,182^ In addition, itaconate has a negative regulatory role in the activation of the NLRP3 inflammasome complex by blocking the interaction between NLRP3 and NEK7 and preventing the induction of dicarboxypropylated C548 on NLRP3.^183^

A recent ex vivo study showed that treatment of macrophages obtained from T2DM patients with the sodium-glucose cotransporter 2 (SGLT2) inhibitor empagliflozin significantly inhibited IL-1β secretion, which was accompanied by increased levels of serum β-hydroxybutyrate (BHB).^184^ Given the inhibitory functions of BHB on NLRP3 inflammasome activity,^185^ these data suggest that NLRP3 inflammasome activation is regulated by ketone bodies. The BHB-induced inhibition of NLRP3 inflammasome activation seems to be mediated through prevention of K^+^ efflux and reduction of ASC oligomerization and speck formation.^185^ Another recent study showed that BHB induces increased expression of the forkhead box O (FOXO) 1 transcription factor and its target gene, heme oxygenase 1, an antioxidative enzyme, thereby providing a protective function against liver injury.^186^ However, the detailed molecular mechanisms by which BHB regulates NLRP3 inflammasome activation remain to be characterized.

In addition, the Src kinase-Cbl pathway has a negative regulatory role in the activation of the NLRP3 inflammasome,^187^ at least partly through the inhibition of GLUT1 expression and decreased glycolytic capacity.^174^ Given that Cbl-b is required for the ubiquitination and proteasomal degradation of NLRP3^41^ and decreases the phosphorylated Pyk2 level,^187^ Cbl may function in multiple ways in the negative regulation of the NLRP3 inflammasome.

β-Glucan-induced immune reprogramming, which is critical for innate immune memory, suppresses ASC oligomerization and speck formation activation in human macrophages to attenuate the NLRP3 inflammasome formation via inhibition of K^+^ efflux and generation of mtROS.^188^ β-glucan-induced memory was beneficial for attenuating IL-1β secretion in macrophages from patients with the NLRP3-associated autoinflammatory disease cryopyrin-associated periodic syndrome (CAPS), suggesting that attenuating IL-1β secretion may have therapeutic potential for NLRP3-related diseases.^188^ It will also be important to determine how innate immune memory affects NLRP3 inflammasome assembly and inhibits activating signals.

##### Autophagy

Autophagy, an intracellular lysosomal degradation pathway, is classified into canonical and noncanonical autophagy pathways.^189–192^ Recent studies have shown that numerous autophagy receptors containing ubiquitin-binding domains and LC3-interacting regions are involved in selective autophagy pathways targeting various types of cargo, including mitochondria, macromolecules such as lipids, aggregated proteins, and intracytoplasmic microbes.^193^ In addition, LC3-associated phagocytosis (LAP) targets phagocytosed particles, such as dying cells or extracellular pathogens.^193–196^ Autophagy acts as the principal inhibitory pathway to limit excessive activation of the NLRP3 inflammasome in the context of various pathological conditions.^189–192^ As numerous reviews have summarized the relationship between autophagy and the inflammasome,^189–192,197,198^ in this section, we describe recent work regarding the mechanisms by which autophagy pathways, in particular autophagy-related genes (ATGs), regulate NLRP3 inflammasome activation and its physiopathological consequences.

The autophagy protein immunity-related GTPase family M protein (IRGM) functions in the regulation of core autophagy machinery by promoting the formation of autophagy initiation complexes.^199^ Recent studies have shown that IRGM interacts with NLRP3 and PYCARD/ASC, thus leading to their autophagic degradation via the Sequestosome1 (SQSTM1)/p62-dependent pathway. IRGM impedes inflammasome assembly by blocking the polymerization of NLRP3 and ASC, thus showing protective effects against intestinal inflammation in a murine DSS-induced colitis model.^200,201^ In addition, aberrant autophagy associated with a truncated UVRAG mutation promotes increased inflammatory responses and colitis-associated tumorigenesis through elevated activation of the NLRP3 inflammasome.^202^ A recent study showed that microglial Atg5 deletion promoted Parkinson’s disease symptoms in a mouse model through upregulation of NLRP3 inflammasome activation via cAMP signaling.^203^ Taken together, these findings strongly suggest that defective expression or dysregulation of ATGs is associated with upregulated NLRP3 inflammasome activation, leading to pathological responses in NLRP3-associated diseases. Notably, in bronchial cells of patients with cystic fibrosis, *Pseudomonas aeruginosa* infection results in impaired autophagy, thereby activating the NLRP3 inflammasome and hyperinflammation in cystic fibrosis pulmonary disease.^204^ Importantly, defective CFTR channels are associated with decreased capacity for selective autophagic clearance of *P. aeruginosa* infection in cystic fibrosis bronchial cells.^204^ The precise mechanism linking CFTR channels to selective autophagy activation remains to be determined. However, it is intriguing to speculate that a persistent mitochondrial unfolded protein response (UPRmt) may be involved in this phenomenon and NLRP3 inflammasome activation bronchial cells in cystic fibrosis.^204^

Several molecules involved in selective autophagy have been reported to play roles in the regulation of the NLRP3 inflammasome. Autophagic adapter SQSTM1-mediated autophagy leads to degradation of pyruvate kinase muscle (PKM), thereby inhibiting the production of mature IL1B in LPS-ATP-treated macrophages and ameliorating synovial inflammation.^205^ PTEN-induced kinase 1 (PINK1)/Parkin-mediated mitophagy suppresses mtROS and NLRP3 inflammasome-related renal injury in renal tubular epithelial cells (RTECs) during contrast-induced acute kidney injury.^206^

Defective mitophagy/autophagy does not always lead to activation of the NLRP3 inflammasome. Deletion of *pink1*, an essential gene for mitophagy, upregulates NLRP3, brown adipose tissue dysfunction, and acquisition of an obesity-prone phenotype, although the canonical NLRP3 inflammasome is not activated.^207^ In addition, autophagic flux and inflammasome activation are linked to the promotion of NLRP3 inflammasome-mediated pathological inflammation induced by 1-deoxysphingolipids (deoxySLs), atypical sphingolipids that are elevated in patients with hereditary sensory and autonomic neuropathy (HSAN1) or T2DM.^208^ Rapidly accumulating evidence regarding the cross talk between autophagy components and the inflammasome that regulates immune responses has provided new insights that are likely to lead to the development of novel therapeutic approaches for treating NLRP3-related diseases.

##### MicroRNAs (miRNAs)

Several studies have confirmed that miRNAs are among the major regulators of the activation of the NLRP3 inflammasome pathways (Table 2).^209^ Among the numerous miRNAs that directly target and suppress NLRP3, miR-223-3p is one of the best studied in terms of inflammasome regulation.^209,210^ Recent preclinical studies support the biological importance of miR-223-3p in the regulation of the NLRP3 inflammasome. For example, the synthetic miR-223 analog miR-223-3p caused remarkable attenuation of inflammation and fibrosis development in mouse models with endotoxin acute hepatitis (EAH) or fibrotic nonalcoholic steatohepatitis (NASH).^211^ Paeonol, a potential therapeutic agent for atherosclerosis, inhibits inflammatory cytokines (IL-1β and IL-6) and NLRP3 inflammasome activation through the upregulation of miR-223 in rat aortic endothelial cells (RAECs).^212^

**Table 2:** Regulatory mechanisms by miRNA and lncRNA upon NLRP3 inflammasome activation

Regulator	NLRP3 regulation	Target	Study model	Associated disease/pathology	Ref.
miR-223	↓	3ʹ-UTR of NLRP3	BMDMs, BMDCs, Neutrophils	–	^210^
			J744.2 macrophages, C57BL/6 mice	EAH and fibrotic NASH	^211^
			RAECs, male SD rats	Atherosclerosis	^212^
miR-139	↓	c-Jun	SH-SY5Y and BV2 cells	Cerebral I/R injury	^213^
miR-183	↓	TXNIP	HEK293T and microglial cells, male SD rats	CCI-induced neuropathic pain	^214^
miR-421	↑	FOXO3a	C2C12 cells, C57BL/6 mice	Ischemic muscle injury	^215^
miR-21	↑	A20	BMDMs, C57BL/6 mice	Septic shock	^216^
LncRNA ADAMTS9-AS2	↑	miR-223-3p	CS-GC and CR-GC cell lines, GC tissues	Gastric cancer	^217^
LncRNA NEAT1	↑	miR-3076-3p	BMDCs, BALB/c mice	Experimental autoimmune myocarditis	^218^

*3*ʹ*-UTR* 3ʹ-untranslated region, *BMDMs* bone marrow-derived macrophages, *BMDCs* bone marrow-derived dendritic cells, *EAH* endotoxin acute hepatitis, *NASH* nonalcoholic steatohepatitis, *RAECs* rat aortic endothelial cells, *SD* Sprague-Dawley, *I/R* ischemia/reperfusion, *TXNIP* thioredoxin interacting protein, *CCI* chronic constriction injury, *FOXO* forkhead box protein O, *CS-GC* cisplatin-sensitive gastric cancer, *CR-GC* cisplatin-resistant gastric cancer

Recent studies have also shown that other miRNAs in addition to miR-223-3p are involved in the negative regulation of the NLRP3 inflammasome. For example, miR-139 targeting c-Jun inhibits nerve injury induced by oxygen-glucose deprivation/reoxygenation (OGD/R) through the inhibition of NLRP3 inflammasome activation and cell pyroptosis.^213^ In addition, miR-183 targeting TXNIP reduces an inflammatory response triggered by the TXNIP-NLRP3 inflammasome, contributing to neuropathic pain in a rat model of chronic constriction injury and in microglia.^214^ In contrast, several miRNAs participate in the induction and activation of the NLRP3 inflammasome, although the precise mechanisms remain to be determined. Studies showed that the repair of ischemic injury by human umbilical cord mesenchymal stem cell-derived exosomes is mediated by targeting the miR-421/FOXO3a pathway, thereby inhibiting NLRP3 inflammasome activation and pyroptosis.^215^ In other words, miR-421 directly targets FOXO3a to upregulate pyroptosis and NLRP3 activation.^215^ The miRNA miR-21 was reported to be a positive regulator of NLRP3 inflammasome activation in myeloid cells through targeting A20, an inhibitor of the NF-κB signaling pathway.^216^ Further studies are warranted to understand the functions of individual miRNAs and the mechanisms underlying their regulatory effects on NLRP3 inflammasome activation under homeostatic, immune, and pathological conditions.

Recent studies have also examined the molecular interplay between long noncoding RNAs (lncRNAs) and miRNAs in terms of NLRP3 inflammasome regulation. The lncRNA ADAMTS9-AS2, a tumor suppressor, enhances cisplatin sensitivity in gastric cancer cells by activating NLRP3-mediated pyroptotic cell death by sponging miR-223-3p.^217^ In addition, knocking down the lncRNA NEAT1 inhibits inflammasome activation through induction of miR-3076-3p targeting NLRP3, thereby expanding the tolerogenic phenotype of dendritic cells.^218^ One important future direction of study involves investigating the mechanisms of cross talk between miRNAs and lncRNAs in the modulation of NLRP3 inflammasome activation and pyroptotic cell death.

##### Hormones and nuclear receptors

There are at least 48 members of the nuclear receptor gene superfamily that regulate a variety of pathophysiological functions, including metabolism, inflammation, and circadian rhythm.^219,220^ A range of nuclear receptors play key roles in the regulation of inflammation and NLRP3 inflammasome activation. A recent study highlighted the circadian oscillation of NLRP3 signaling activation and indicated that the circadian clock is essential for the inhibition of inflammation and optimal activation of the NLRP3 inflammasome.^221^ Recent data strongly suggest the potential benefit of chronotherapy in the pathology of dysregulated NLRP3 signaling activation.^221^ In support of this report, the core clock component nuclear receptor subfamily 1 group D member 1 (NR1D1, also called Rev-erbα) was shown to be essential for controlling the activity of the NLRP3 inflammasome pathway. Recent studies in Nr1d1-deficient mice showed that NR1D1 is required for the regulation of NLRP3 expression and activation, thereby inhibiting peritoneal inflammation and fulminant hepatitis.^222^

Several studies have reported negative regulatory functions of nuclear receptors in terms of the NLRP3 inflammasome pathway impacting, in particular, the priming step of NLRP3 activation. Small heterodimer partner (SHP), an orphan nuclear receptor, physically interacts with NLRP3 and suppresses activation of the NLRP3 inflammasome.^223^ Nuclear receptor related 1 (Nurr1/NR4A2) ameliorates the activation of Müller cells and the cell death of retinal ganglion cells in a diabetes model through suppression of NF-κB action and inhibition of NLRP3 inflammasome component expression, such as NLRP3 and ASC.^224^ However, some nuclear receptors may function in the activation of the NLRP3 inflammasome. All-*trans*-retinoic acid, a derivative of vitamin A, induces the expression of NLRP3 and pro-IL-1β at the priming step and promotes activation of the NLRP3 inflammasome by inducing human macrophages to undergo glycolysis.^225^ Further investigations of nuclear receptor interactions with NLRP3 inflammasome pathway components are likely to provide an explanation for the molecular mechanisms underlying the priming step being regulated in a gene-specific manner.

A recent study showed that the antifibrotic hormone relaxin attenuates profibrotic TGF-β1/IL-1β signaling through inhibition of TLR4-dependent priming in NLRP3 inflammasome activation.^226^ Although another study showed that the antifibrotic activity of relaxin is mediated by targeting caspase-1 in human dermal fibroblasts,^227^ it is unclear whether relaxin directly inhibits caspase-1 activity or whether it attenuates assembly of the NLRP3 inflammasome complex. There is a continuing need to accumulate data to investigate the functions of a variety of hormones and their receptors in the regulatory effects induced upon NLRP3-induced pathologies.

##### Others: cytokines, adapters, Notch1, cAMP, Foxp1, etc.

A variety of cytokines, signaling molecules, and second messengers are potentially involved in the positive or negative regulation of NLRP3 inflammasome activation. Recent studies have shown that IL-37d, a newly discovered negative immune regulator, inhibits the priming step of NLRP3 expression through suppression of NF-κB signaling activation. IL-37d transgenic mice show increased resistance to DSS-induced acute colitis and inhibition of NLRP3 inflammasome overactivation.^228^ In addition, peritoneal tissue-resident macrophages lacking the tissue-specific transcription factor GATA6 robustly suppressed IL-1β processing through the action of Gata6-mediated production of prostacyclin and IL-10.^229^ Further studies will be needed to evaluate the regulatory effect of a wide range of cytokines on NLRP3 inflammasome activation.

Several adapter molecules, the functions of which were identified in immune cell signaling, have been suggested to significantly fine-tune NLRP3 inflammasome activation. For example, B cell adapter for phosphoinositide 3-kinase (PI3K) (BCAP) and its association with interacting proteins, such as the caspase-1 pseudosubstrate inhibitor Flightless-1, delays the recruitment of procaspase-1 within the NLRP3–ASC preinflammasome, thereby inhibiting the activation of the NLRP3 inflammasome in macrophages.^230^ The Toll-IL-1R protein SARM regulates cell survival and IL-1β release upon inflammasome activation by increasing inflammasome-dependent IL-1β production and reducing pyroptosis when SARM is removed from macrophages.^231^ Moreover, SARM-mediated mitochondrial depolarization determines whether pyroptosis occurs in cells after NLRP3 inflammasome activation.^231^

Several established signaling molecules, including Notch1, cAMP, and Foxp1, play crucial negative roles in the regulation of the NLRP3 inflammasome in immune cells. Jagged1 (JAG1)-mediated Notch1 signaling in myeloid cells upregulates heat shock transcription factor 1 (HSF1) expression and Snail activity to control NLRP3/caspase-1 activity.^232^ As discussed in the section on Ca^2+^ flux, binding of cAMP to NLRP3 leads to inhibition of inflammasome assembly.^105^ Activation of the cAMP-PKA signaling pathway is linked to inhibition of NLRP3 inflammasome activity through enhancement of K63-linked ubiquitination of NLRP3.^233^ Genistein-mediated anti-inflammasome activity is mediated through TGF5-cAMP signaling via increased intracellular cAMP levels^234^ Foxp1 was reported to have a negative regulatory function on endothelial NLRP3 inflammasome activation, acting as a gatekeeper of vessel inflammation.^235^ Endothelial Foxp1 is regulated by Krüppel-like factor 2 (Klf2) and further regulates NLRP3 inflammasome activation through direct regulation of endothelial inflammasome components, including NLRP3 and caspase-1.^235^ Exploring the effects of a variety of signaling molecules and/or second messengers on inflammasome regulation may lead to the discovery of potential therapeutic targets against NLRP3-related pathologic inflammation.

Cellular inhibitor of apoptosis protein (cIAP) 1 and cIAP2, members of the IAP family, act as E3 ligases and modulators of the NLRP3 inflammasome.^236,237^ Upon overexpression of cIAP1 or cIAP2 in macrophages, the levels of IL-1β and pyroptotic cell death are increased in response to inflammasome activators or bacterial infections. Glomulin (GLMN), originally identified through its association with glomuvenous malformations, acts as an inhibitor of Cullin-truly interesting new gene (RING)-box protein 1 (RBX1) E3 ligases and binds to the RING domains of cIAP1 and cIAP2, thereby inhibiting their functions.^238^

On the other hand, the human serum factor H-related protein FHR1 binds to necrotic cells via its N-terminus and upregulates NLRP3 inflammasome activation in human monocytes, thereby producing IL-1β, TNFα, IL-18, and IL-6, thus contributing to the pathology of anti-neutrophil cytoplasmic antibody-associated vasculitis (AAV) and atherosclerosis.^239^ Recent studies have also shown that monoamine oxidase (MAO) catalyzes the oxidative deamination of neurotransmitters and amines, generating mtROS and NLPR3 inflammasome activation through a NF-κB-mediated mechanism.^240^ A number of mechanisms remain to be addressed before we can gain a full understanding of the multiple molecules/pathways that positively and negatively regulate NLRP3 signaling networks in immune cells.

#### Small molecules/agents as therapeutics against NLRP3 inflammasome activation

There has been rapid progress in the identification of NLRP3 inflammasome-targeting small molecules/agents for use as therapeutics against NLRP3 inflammasome activation. Accumulating evidence has revealed large numbers of inhibitors of NLRP3 inflammasome activation through various pharmacological approaches used with NLRP3 inflammasome-related disease models.^13,241,242^ Several extensive reviews summarizing NLRP3 inflammasome activators and inhibitors have suggested that developing new small molecules that directly target NLRP3 seems to be more specific, cost-effective, and safer than an overall cytokine blockade.^241,242^ Determination of the complexity of the NLRP3 inflammasome structure and interactions among its components holds promise for the development of new molecules targeting specific components or interactions of the NLRP3 inflammasome complex. Here, we briefly describe the most potent and most recently discovered inhibitors according to their known targets (Table 3).

**Table 3:** Small molecules/agents as therapeutics against NLRP3 inflammasome activation

Small molecule/ agent	Chemical class	Interaction with target	Ref.
Targeting NLRP3 NACHT domain			
MCC950/CRID3	Diarylsulfonylurea compound	Binds to Walker B motif of NACHT ATPase	^243^
CY-09	CFTR_(inh)_-172 analog	Binds to Walker A motif of NACHT ATPase	^249^
OLT1177	β-sulfonyl nitrile compound	Directly targets ATPase and inhibits NLRP3 inflammasome oligomerization	^251^
BOT-4-one	Benzoxathiole derivative	Alkylates NLRP3 to impair ATPase activity	^252^
Tranilast	Tryptophan metabolite	Binds to NACHT domain and inhibits NLRP3 oligomerization	^254^
Targeting NEK7-NLRP3 interaction			
Oridonin	Diterpenoid purified from *Rabdosia rubescens*	Irreversibly binds to NLRP3 Cys279 and inhibits NLRP3–NEK7 interaction	^255^
Rg3	Ginsenoside extracted from *Panax ginseng*	Abrogates NEK7-NLRP3 interaction, and subsequently inhibits NLRP3-ASC interaction, ASC oligomerization, and speckle formation	^256^
C1-27 (& 25)	Benzenesulfonamide derivative	Inhibits GSTO1-1, which is NEK7 deglutathionylating enzyme	^257,258^
Artemisinin	Sesquiterpene lactone isolated from *Artemisia annua*	Targets and inhibits interaction between NEK7 and NLRP3	^259^
Targeting PYD			
KN3014	Piperidine-containing compound	Directly targets PYD and inhibits the interaction between NLRP3 and ASC	^260^
β-carotene	Provitamin A carotenoid	Binds to NLRP3 PYD	^261^
ASC^PYD/H2-H3^ peptide	Peptide corresponding to H2-H3 segment of ASC PYD	Binds to NLRP3 PYD and selectively inhibits NLRP3 inflammasome	^262^

The NACHT domain of NLRP3 is the molecular target of diarylsulfonylurea inhibitors, including MCC950/CRID3,^243^ which is a potent and selective inhibitor of the NLRP3 inflammasome pathway through its interaction with the Walker B motif within the NACHT domain of NLRP3 by which ATP hydrolysis is blocked.^244^ The mechanism of action of MCC950 is mediated by changing the active conformation of NLRP3 into an inactive state.^245^ NLRP3 inhibition with MCC950 was shown to significantly suppress IL-1β production and airway inflammation in the lungs of mice with cystic fibrosis^246^ and to prevent cognitive deficits in mice with experimental autoimmune encephalomyelitis (EAE).^247^ However, recent studies have shown that MCC950/CRID3 targets wild-type NLRP3 but not NLRP3 gain-of-function point mutants related to CAPS.^248^ The CFTR_(inh)_-172 analog CY-09 also inhibits NLRP3 ATPase activity by directly binding to the ATP-binding motif of the NLRP3 NACHT domain.^249^ Prominent therapeutic effects were observed in mouse models of CAPS and T2DM and in monocytes of gout patients^249^ treated with CY-09. A recent study showed that CY-09 treatment is beneficial in ameliorating epileptic progression and neuronal loss through attenuation of NLRP3-dependent IL-1β secretion and astrocyte activation.^250^ Moreover, a β-sulfonyl nitrile compound, OLT1177, reduces ATPase activity by directly binding to NLRP3, followed by inhibition of ASC speck aggregation,^251^ and BOT-4-one impairs NLRP3 ATPase activity by alkylating NLRP3, leading to obstruction of NLRP3 inflammasome assembly.^252^ Tranilast, a tryptophan metabolite used for the treatment of allergies and asthma,^253^ shows remarkable preventive and therapeutic effects in mouse models of gout, CAPS, and T2DM by hindering NLRP3 oligomerization in an ATPase-independent manner.^254^

With evidence of the importance of NEK7, there is increasing interest in discovering new drugs targeting NEK7 and its interaction with NLRP3, as most of the small molecules targeting the NLRP3 inflammasome were reported prior to the publication of the cryoelectron microscopy structure of NEK7–NLRP3.^10^ Oridonin, which is the main ingredient of the traditional Chinese herbal medicine *Rabdosia rubescens*, blocks the interaction between NLRP3 and NEK7 by forming a covalent bond with cysteine 279 in the NACHT domain.^70,255^ Oridonin shows both preventive and therapeutic effects in peritonitis, gouty arthritis, and T2DM mice via inhibition of NLRP3 activation.^255^ Ginsenoside Rg3, a natural product extracted from *Panax ginseng*, was recently reported to selectively inhibit NLRP3 activation. Rg3 does not regulate the upstream signals of the NLRP3 inflammasome but mechanistically abrogates the NEK7–NLRP3 interaction, thereby subsequently disturbing NLRP3–ASC assembly.^256^ Hughes et al. reported that deglutathionylation of NEK7 by glutathione transferase omega 1-1 (GSTO1-1), a constitutive deglutathionylating enzyme, is required for activation of the NLRP3 inflammasome.^257^ They used the GSTO1-1 inhibitor C1-27 to show that inhibition of GSTO1-1 had a protective effect in an ECE mouse model, and a more advanced form of inhibitor, designated 25, was reported in a follow-up study.^258^ In addition, artemisinin^259^ targeted NEK7–NLRP3 interactions to suppress inflammasome activity in a T2DM disease model.

Studies of small compounds targeting the NLRP3 inflammasome identified KN3014, which directly targets the PYD and thus inhibits the interaction between NLRP3 and ASC.^260^ KN3014 was shown to block ASC speck formation effectively and significantly reduced IL-1β secretion from the PBMCs of a patient with Muckle–Wells syndrome (MWS). Another study with β-carotene (provitamin A) demonstrated its direct binding to the PYD of NLRP3,^261^ inhibiting IL-1β secretion from synovial fluid cells retrieved from patients with gouty arthritis. Recent studies identified several peptides that modulate different stages of NLRP3 inflammasome assembly and inhibit IL-1β release, caspase-1 activation, and ASC oligomerization.^262^ Among these candidates, a peptide with a sequence corresponding to the H2-H3 segment of ASC PYD showed selective inhibitory activity against NLRP3 but was not absent in melanoma 2 (AIM2) and NLR family CARD domain-containing protein 4 (NLRC4) inflammasomes.^262^

There is a great deal of research interest in the inflammasome. A wide range of natural and synthetic inhibitors have been reported to have inflammasome-inhibiting activity. However, insufficient understanding of the mechanisms of action and potential off-target effects of these molecules limit their further development for clinical use. With the elucidation of the structure and mechanism of inflammasome formation, the identification and design of new inhibitors targeting specific components of the NLRP3 inflammasome will provide new insights and facilitate the development of therapeutics for various autoinflammatory and autoimmune diseases.

## Conclusion

The study of NLRP3 inflammasome activation has many implications for health and disease. Significant progress has been made toward understanding the molecular mechanisms underlying the priming/licensing step of NLRP3 inflammasome activation. However, many questions remain, e.g., how the individual and/or multiple PTM regulation is curated for licensing of the NLRP3 inflammasome, how different NLRP3 stimuli are engaged to converge into the signaling cascades that lead to full activation of the NLRP3 inflammasome, and which signaling pathway(s) play key roles in the ultimate assembly of the inflammasome complex. The spatiotemporal regulation of NLRP3 inflammasome activation has not yet been completely elucidated, but recent studies have highlighted the emerging concept of the involvement of the dispersed Golgi apparatus and MTOC in the formation of the NLRP3 inflammasome complex. Further identification of key players involved in regulating the stepwise engagement of organelles and sequential interorganellar communication is needed to clarify the molecular mechanisms of NLRP3 inflammasome activation. It remains to be determined how multiple positive/negative regulatory mechanisms are involved in NLRP3 inflammasome activation as well as the nature of their cell type-specific and NLRP3 stimulus-specific roles. Moreover, the roles of immunometabolism, miRNAs, and lncRNAs in NLRP3 inflammasome activation have only begun to be elucidated. A wide range of small molecules/reagents are being developed for the regulation of NLRP3 inflammasome activation, although therapeutic outcomes have been limited in terms of clinical trials. Future work may provide a comprehensive interpretation of the highly regulated nature of the formation and activation of the NLRP3 inflammasome complex. These efforts will be critical for the future development of potential therapeutic and preventive agents for NLRP3-related diseases.
